# Oat protein nanofibril–iron hybrids offer a stable, high-absorption iron delivery platform for iron fortification

**DOI:** 10.1038/s43016-025-01260-6

**Published:** 2025-11-10

**Authors:** Jiangtao Zhou, Sueppong Gowachirapant, Christophe Zeder, Alexander Wieczorek, Jeannette Nuessli Guth, Ines Kutzli, Sebastian Siol, Ferdinand von Meyenn, Michael B. Zimmermann, Raffaele Mezzenga

**Affiliations:** 1 Laboratory of Food and Soft Materials, Department of Health Sciences and Technology, ETH Zurich, Zurich, Switzerland; 2 Institute of Nutrition, Mahidol University, Nakhon Pathom, Thailand; 3 Laboratory of Clinical Biopharmacy, Department of Chemistry and Applied Biosciences, ETH Zurich, Zurich, Switzerland; 4 Laboratory for Surface Science and Coating Technologies, Empa – Swiss Federal Laboratories for Materials Science and Technology, Duebendorf, Switzerland; 5 Consumer Behavior, Department of Health Sciences and Technology, ETH Zurich, Zurich, Switzerland; 6 Laboratory of Nutrition and Metabolic Epigenetics, Department of Health Sciences and Technology, ETH Zurich, Zurich, Switzerland; 7 Department of Medical and Molecular Genetics, King’s College London, London, UK; 8 Translational Immunology Discovery Unit, MRC Weatherall Institute of Molecular Medicine, John Radcliffe Hospital, University of Oxford, Oxford, UK; 9 Department of Materials, ETH Zurich, Zurich, Switzerland; 10 Present Address: Department of Food Science and Technology, National University of Singapore, Singapore, Singapore

**Keywords:** Risk factors, Metabolism, Proteins

## Abstract

Iron deficiency and anaemia affect two billion people globally. Iron fortification can help to treat anaemia, but most current fortificants are limited by low absorption and/or poor sensory properties. Here we introduce oat protein nanofibrils (OatNF) carrying ultrasmall iron nanoparticles that are engineered to carry iron in ferrous or ferric form. In a prospective cross-over stable-isotope absorption trial in young iron-deficient women (*n* = 52), OatNF reduced with sodium ascorbate carried mainly ferrous iron and showed high fractional absorption with water and with polyphenol-rich food, showing 1.76- and 1.65-fold higher absorption, respectively, compared with ferrous sulfate. When sodium hydroxide was used as the reducing agent, OatNF carried mainly ferric iron, which was also well absorbed and featured good sensory properties in reactive food matrices. OatNF hybrids offer a plant-based strategy for delivering highly bioavailable iron for food fortification.

## Abstract (web summary)

Nanofibrils constructed from oat protein and carrying iron nanoparticles can increase absorption compared with ferrous sulfate in human studies, offering a promising fortification strategy for treating iron deficiency and improving human nutrition.

## Main

The worldwide prevalence of anaemia across all age groups is 24.3% (95% uncertainty interval 23.9–24.7), affecting approximately 1.92 billion people^1–4^. Dietary iron deficiency is the leading cause of anaemia-related years lived with disability, with a rate of 422.4 years lived with disability (95% uncertainty interval 286.1–612.9) per 100,000 population^1^. To reduce anaemia, the World Health Organization (WHO) recommends use of ferrous iron salts for fortification of most foods^5^, but most iron fortificants remain poorly effective due to low bioavailability and poor sensory performance^2,3,6–9^. Therefore, developing a robust technology capable to deliver highly bioavailable iron in foods without causing negative sensory effects is still highly sought after^8,10,11^. For example, although the WHO has set a global nutrition target to reduce anaemia prevalence by 50% among women of reproductive age (15–49 years) by 2030 (ref. ^12^), the global prevalence of anaemia in this group of women remains stubbornly high, at 30% (27–33%)^13^. Symptoms of anaemia in women include fatigue, weakness, difficulty concentrating, dizziness, pallor and headache^8^.

The recommended daily iron intake for young women is 18 mg (ref. ^14^), but this high requirement is most often not met through diet alone. To address this, iron fortification of staple foods is the recommended strategy to increase iron intake among young women^5,6^. The WHO recommends use of ferrous iron salts for fortification of most foods, with ferrous sulfate (FeSO_4_) considered the gold standard due to its high bioavailability and low cost^5^. However, incorporating ferrous iron into many foods is challenging because it typically causes undesirable sensory changes^5^. Furthermore, iron added to food is often poorly absorbed because of inhibitory compounds, particularly polyphenols and phytic acid^6^. Low iron absorption from fortificants also results in large amounts of unabsorbed luminal iron that may provoke gut inflammation and dysbiosis^15,16^. Finally, high amounts of absorbed iron may generate non-transferrin-bound iron, which may be pro-oxidant and toxic. To partially address this, compounds such as sodium iron EDTA (NaFeEDTA) have been developed, offering better absorption compared with ferrous salts when used in food matrices with inhibitory compounds^5^. In addition, ferrous bisglycinate is an aminochelated iron fortificant with proven efficacy in specific applications such as milk, but the WHO does not recommend it for large-scale food fortification owing to its high cost and sensory issues in foods^6^. Thus, despite these advances, finding a method to deliver highly bioavailable iron in foods without causing negative sensory effects is still highly sought after, and remains both a major challenge and a key area of research.

Here, we introduce oat protein nanofibrils (OatNF) carrying ultrasmall iron nanoparticles as a promising strategy to provide highly bioavailable iron with enhanced sensory performance in foods and beverages. We show that, when sodium ascorbate (SA) is used as a reducing agent of iron salt precursors in the presence of OatNF, subnanometre nanoparticles of ferrous iron form on the surface of the OatNF (OatNF-SA-Fe), producing hybrids with superior iron delivery features. OatNF exhibited strong iron-binding, reducing and stabilizing properties during the synthesis of OatNF-SA-Fe hybrids, resulting in exceptional ferrous iron stability.

In a prospective cross-over study in iron-deficient Thai women (*n* = 52), we used erythrocyte incorporation of oral stable iron isotopes^17^ to quantify the iron absorption and bioavailability from OatNF-SA-Fe hybrids. Each iron compound provided a dose of 4 mg of elemental iron in both the water and meal conditions; this dose was selected as it is representative of typical fortification levels. Absorption from the hybrids was quantified when given with water alone and with a highly inhibitory polyphenol-rich meal, and compared with FeSO_4_.

The OatNF-SA-Fe hybrids were highly absorbed, with a geometric mean absorption rate of 46.2% (95% confidence interval (CI) 38.9–55.0%) when administered with water, 76% higher than absorption from FeSO_4_, which had an absorption rate of 26.3% (95% CI 21.4–32.4%). Similarly, when administered with a polyphenol-rich meal, the iron absorption from the OatNF-SA-Fe hybrids was 65% greater than from FeSO_4_. We infer that the combination of OatNF and SA (at a 2:1 molar ratio to iron) enhances iron absorption, probably due to their reducing and stabilizing effects, as well as the relatively high content of glutamine in the OatNF. This conclusion is further supported by experiments using NaOH-reduced hybrids (OatNF-NaOH-Fe), which, in contrast to the OatNF-SA-Fe hybrids, contain high ferric iron content. These hybrids were also well absorbed and achieved 77% and 75% bioavailability compared with FeSO_4_ when administered with water and a polyphenol-rich meal, respectively. In addition, the OatNF-Fe hybrids demonstrated excellent organoleptic qualities, showing minimal sensory impact when added to common foods and beverages.

These findings build on our previous research, in which we first proposed the use of nanosized iron as an iron fortification strategy^18^ and showed that nanosized ferric phosphate is well absorbed in both mice and humans^19^. Yet, in those studies, iron bioavailability remained lower than that of FeSO_4_, and, in humans, achieved only 72% relative bioavailability to FeSO_4_. We also demonstrated the high bioavailability of iron nanoparticle–milk-derived protein nanofibrils in rats^20^ and confirmed the safety of food amyloid fibrils as nutritional ingredients through in vitro and in vivo assessments^21^. Although these nanofibril studies were limited to mouse models and used animal-derived proteins, they provide the foundation for the present work, in which iron bioavailability in humans reached 176% relative to FeSO_4_. Given the above, we believe that the plant-based OatNF-SA-Fe hybrids introduced in this work may offer a breakthrough strategy for effective delivery of highly absorbed iron in fortified foods and beverages and may contribute to the mitigation of iron deficiency and anaemia on a global scale.

## Results

### The fabrication of the OatNF-SA-Fe hybrid

**Fig. 1:**
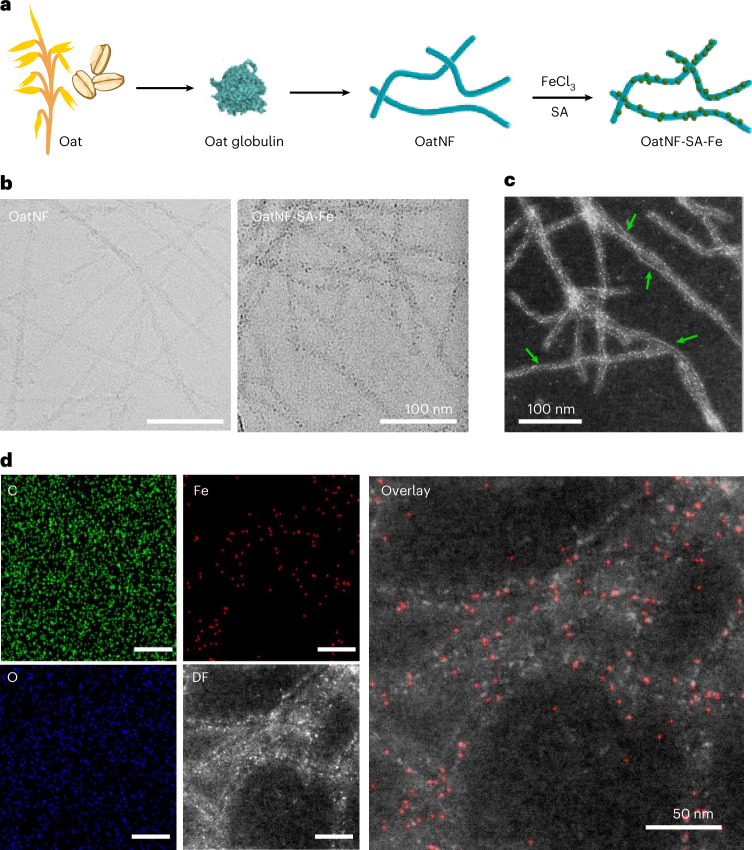
Fabrication and electron microscopy characterization of the OatNF-SA-Fe hybrid. **a**, A schematic illustration of the fabrication process of the OatNF-SA-Fe hybrid. The illustration of monomeric OG is based on UniProt P12615. **b**, Cryo-electron microscopy images of oat nanofibril and OatNF-SA-Fe hybrids before and after iron nanoparticle synthesis. **c**, HAADF-STEM image of the OatNF-SA-Fe hybrid, where the bright dots anchored and necklace-aligned (arrow) along OatNF correspond to the heavier atomic number of iron in the hybrid. **d**, EDS elemental mapping images of the OatNF-SA-Fe hybrids, showing distributions of carbon (C), oxygen (O) and iron (Fe) and the corresponding dark field (DF) HAADF-STEM image. The overlay of the HAADF-STEM image and Fe elemental map confirms the accumulation of iron along the oat nanofibrils.

The preparation process of the OatNF-SA-Fe hybrid is shown in Fig. 1a. Initially, oat globulin (OG), the primary oat protein, was extracted from oat flake powder and then incubated to fabricate OG nanofibrils under acidic conditions (pH 2) and heat treatment^22^. The colloidal dispersion of OG nanofibril was subsequently mixed with iron chloride and SA, yielding a translucent dispersion of iron–nanofibril (OatNF-SA-Fe) hybrids.

Cryo-transmission electron microscopy (cryo-TEM) images (Fig. 1b and Supplementary Fig. 1a) revealed that iron nanoparticles formed and were anchored onto the surface of OG nanofibrils. This iron decoration was more evident using high-angle annular dark field scanning TEM (HAADF-STEM) images (Fig. 1c and Supplementary Fig. 1b). Numerous bright dots are visible along the oat nanofibrils, representing iron particles. These are attributed to atomic number contrast between iron and the amino acid elements in the protein nanofibrils. Given the observed diameter of 4–10 nm for the OatNF, these iron particles were estimated to be at most 1 nm or less in size. This nanoscale size is probably due to ascorbate-mediated iron chelation that prevents iron aggregation into large complexes^23^. Remarkably, these subnanometre particles aligned along the fibrils (Fig. 1c and Supplementary Fig. 1b). The presence and distribution of iron on these nanoparticles are further validated by elemental mapping using energy-dispersive X-ray spectroscopy (EDS) analysis (Fig. 1d and Supplementary Fig. 1c). The overlay of the Fe elemental map and the corresponding HAADF-STEM image demonstrated the iron conformation in these subnanometre particles along the surface of OatNF.

To prove the role of SA–Fe chelation in forming subnanometre iron particles, we conducted a control study by mixing OatNF with FeCl_3_ and sodium hydroxide (NaOH). Cryo-TEM and HAADF-TEM images (Supplementary Fig. 2) showed the formation of iron nanoparticles with sizes in the tens of nanometres, consistent with our previous study^24^. These particles were significantly larger than those in the OatNF-SA-Fe hybrid, indicating that NaOH-reduced iron tended to aggregate into large complexes before nucleating on the OatNF surface and forming the OatNF-NaOH-Fe hybrid. Furthermore, Fourier transform infrared spectroscopy also confirmed SA–iron chelation during the formation of iron particles in the OatNF-SA-Fe hybrid.

### Chemo-physical characterization of the OatNF-SA-Fe hybrid

The morphological and chemo-physical characterization of the iron complex in the OatNF-SA-Fe hybrid was carried out to evaluate the conversion rate of Fe(III) into Fe(II). Three complementary methodologies were used to assess this conversion across different length scales.

**Fig. 2:**
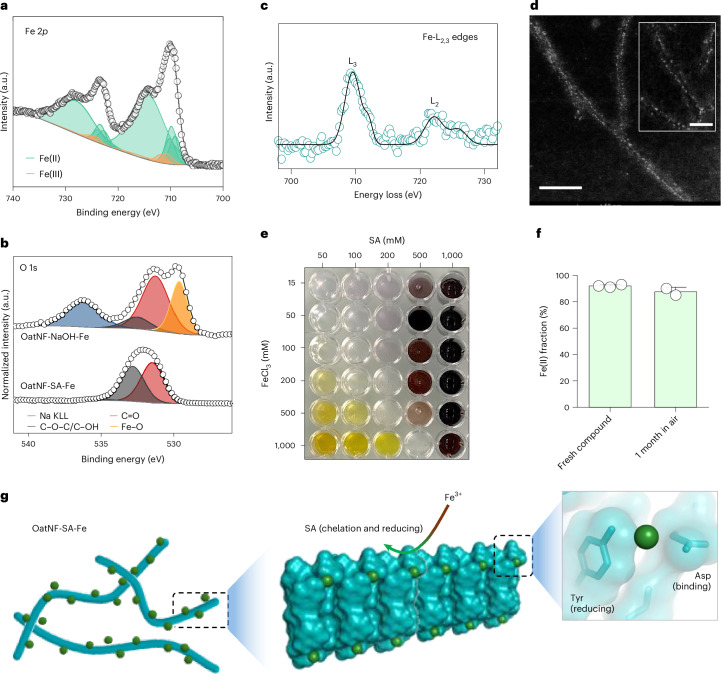
Characterization of the OatNF-SA-Fe hybrids. **a**,**b**, The XPS spectrum of the representative Fe 2*p* region (**a**) for the OatNF-SA-Fe hybrid. Multicomponent fitting was performed according to previous literature^25^. The XPS spectrum of the O 1*s* region (**b**) comparing the OatNF-SA-Fe and OatNF-NaOH-Fe hybrids. The Fe–O feature is clearly visible in OatNF-NaOH-Fe hybrid but is absent in the OatNF-SA-Fe hybrid. **c**, Background-subtracted EELS spectrum of the OatNF-SA-Fe hybrid at the FeL_2,3_ edges. **d**, HAADF-TEM image of aligned iron particles on the nanofibril surface in the OatNF-SA-Fe hybrid. Scale bars, 50 nm. **e**, OatNF-SA-Fe dispersion at different concentrations of FeCl_3_ and SA. **f**, The fraction of ferrous iron in freshly prepared OatNF-SA-Fe hybrid (*n* = 3) and after exposure to air for 1 month (*n* = 2), demonstrating the stability of the ferrous state. Plot is shown as mean ± s.d. **g**, Illustration of chelation, binding, reduction, stabilization and preservation of iron particles (green) on the OatNF surface. The X-ray crystallographic structure of the amyloid-forming fragment (Ala–Val–Try–Val–Phe–Asp) of oat protein reveals that surface amino acids (AAs), including aspartic acid and tyrosine, in OatNF may serve as specific sites for iron binding and reduction, respectively. Panel **g** created with PyMOL 3.1. Source data

X-ray photoelectron spectroscopy (XPS) was used to determine the chemical state of OatNF-SA-Fe. Figure 2a shows the XPS Fe 2*p* spectrum of the OatNF-SA-Fe hybrid peaking at 710 and 715 eV. Remarkably, the O 1*s* spectrum (Fig. 2b and Supplementary Fig. 3c) indicates the absence of Fe–O bonds, associated with iron oxides, iron hydroxides and oxygen ligands. Aside from Na and Cl, no other contamination was found in OatNF-SA-Fe compound (Supplementary Fig. 3a) indicating that iron is primarily present as non-oxide Fe(II) and Fe(III) species. Fitting of the Fe 2*p* region (Fig. 2a and Supplementary Fig. 3c) revealed a remarkably high amount (~91 ± 5%) of bioavailable Fe (II) in the OatNF-SA-Fe hybrid. This high conversion rate is attributed to the subnanometre iron particles anchored on the OatNF surface that can be easily reduced by protein nanofibrils^20^. By contrast, the OatNF-NaOH-Fe hybrid showed larger iron complexes on the fibril surface (Supplementary Fig. 2c), and its O 1*s* spectra (Fig. 2b and Supplementary Fig. 3d) indicated a clear Fe–O feature. Fitting of the Fe 2*p* region (Supplementary Fig. 3d), based on peak constraints^25^, indicates that approximately 30–40% of the iron is present as Fe(II), with the remaining 60–70% as Fe(III), consistent with our previous study^24^.

Electron energy loss spectroscopy (EELS) coupled with STEM provides microscale insights into the Fe valence state in the OatNF-SA-Fe hybrid. The EELS spectrum at Fe L_2,3_ edges (Fig. 2c and Supplementary Fig. 4p) displayed two peaks corresponding to L_3_ and L_2_ edges with a line separation of 13.2 eV. The Fe L_3_ edge exhibited two subpeaks: a main peak at 709 eV and a minor shoulder at 711 eV, corresponding to ferrous and ferric content, respectively^26,27^. The EELS L_3_/L_2_ intensity ratio^28^ is related to the Fe valence state (Fe^3+^/∑Fe)^26,29^, revealing only 11.7% total iron in the ferric state (Fe³⁺). The HAADF-STEM images (Fig. 2d) demonstrated that rich iron complexes were well aligned along the OatNF surface, onfirming specific OatNF–iron chelation that probably facilitates the reduction of subnanometre iron complexes to a high ferrous content.

The iron valence state was further investigated using a bulk colorimetric assay. Dispersions with varying SA-to-Fe ratios (Fig. 2e) appeared yellow when FeCl_3_ was in excess, mahogany with excess SA due to the formation of large insoluble complexes^30^ and translucent at an SA-to-Fe ratio of approximately 2:1. We applied colorimetric assay to measure the Fe^2+^/Fe^3+^ ratio (Supplementary Fig. 4a–e), indicating that extreme SA-to-Fe ratios resulted in low Fe^2+^/Fe^3+^ ratios due to SA–Fe chelation or SA depletion. Remarkably, the optimized SA-to-Fe ratio of 2:1 achieved a high Fe^2+^/Fe^3+^ ratio of 5.6:1 (85% Fe^2+^). We further investigated the ferric and ferrous content via colorimetric assay using ferrozine and ammonium thiocyanate (Supplementary Fig. 4f–o), confirming the high ferrous content in OatNF-SA-Fe dispersion.

The synthesis of the OatNF-SA-Fe hybrid is illustrated in Fig. 2g. An appropriate SA concentration enables mild SA–iron chelation^23^ forming subnanometre iron particles that bind to OatNF. The alignment of subnanometre particles (Figs. 1c,d and 2d and Supplementary Fig. 1b) suggests specific iron-binding sites on the OatNF surface. These sites are probably the carboxyl groups of glutamic and aspartic acids^31,32^, which are abundant in OG (Supplementary Fig. 4q), forming stable tridentate iron complexes^32,33^. Subsequently these immobilized iron particles are reduced by amino acids on the nanofibril surface, including cysteine, tryptophan and especially tyrosine^20^, which is abundant in oat protein. Figure 2g also highlights the amyloid-forming fragments (Ala–Val–Try–Val–Phe–Asp) in oat protein, illustrating that aspartic acid facilitates iron binding and tyrosine contributes to iron reduction. This chelation–binding–reduction process achieves a 90% ferric-to-ferrous conversion. Furthermore, OatNF exhibits strong antioxidant activities, effectively stabilizing and preserving bioavailable ferrous complexes. The freeze-dried OatNF-SA-Fe retained a high ferrous content after 1 month of air exposure (Fig. 2f and Supplementary Fig. 3e), demonstrating its exceptional long-term stability for practical applications in the iron fortification of foods.

### Complete digestion in gastrointestinal conditions

The safety of OatNF-SA-Fe and OatNF-NaOH-Fe hybrids is supported by extensive literature and recent findings. Hilty et al.^18^ demonstrated that nanosized iron as a food ingredient is safe, reporting no adverse effects in mice. Baumgartner et al.^19^ further confirmed its safety and high bioavailability in a clinical trial involving iron-deficient anaemic women.

Iron–protein nanofibril hybrid materials have also been shown to be safe. Shen et al.^20^ provided strong evidence that iron oxide nanofibril hybrids fully dissolve during digestion, both in vitro and in vivo. Using small-angle neutron scattering with contrast matching, they showed that the iron nanoparticles completely dissolve in the stomach. Histological analysis in mice fed iron nanofibrils revealed no accumulation or uptake of intact nanoparticles—an important finding that indicates these particles do not persist in the body or pose a risk of organ toxicity. Xu et al.^21^ further demonstrated, through both cell culture and animal models, that protein nanofibrils are biocompatible and do not induce adverse effects. While earlier studies focused on milk- and egg-derived nanofibrils, this study uses OG nanofibrils and iron nanoparticles synthesized via a slightly modified reduction process. To address the impact of these modifications, we conducted an in vitro digestion study using the standardized INFOGEST protocol^34^ (Supplementary Fig. 5). The results align with previous findings by Xu et al.^21^ and Shen et al.^20^ where oat nanofibrils were substantially degraded in the gastric phase due to pepsin hydrolysis and were completely digested during the intestinal phase, as observed by Xu et al.^21^. The iron nanoparticles, consistent with Shen et al.^20^, were fully dissolved in the stomach phase, as confirmed by atomic force microscopy. In conclusion, the cumulative evidence from prior studies^18–21^, together with new digestion data based on the INFOGEST protocol (Supplementary Fig. 5), supports the safety profile of both OatNF-SA-Fe and OatNF-NaOH-Fe hybrids.

### Sensory study in fortified foods and beverages

We next assessed the sensory performance of the OatNF-SA-Fe and OatNF-NaOH-Fe hybrids compared with several commercial compounds when added to three food and beverage matrices (water, apple juice and cow’s milk yogurt 1.1% fat). To quantify their sensory impact, we first measured the colour changes induced by the various iron compounds 2 h after their addition to the three matrices. All compounds were added to the food and beverages at a fortification level of 2.5 mg Fe per 100 g of matrix. As our comparators to the OatNF-SA-Fe and OatNF-NaOH-Fe compounds, we chose ferrous sulfate and ferrous fumarate, the two main iron compounds recommended by the WHO for the iron fortification of foods^5^. These iron compounds, although highly bioavailable, often cause colour and/or flavour changes in foods. As our ‘negative’ comparators, we chose ferric pyrophosphate and ferrous bisglycinate, relatively non-reactive iron compounds used to fortify some foods that are particularly colour sensitive.

**Fig. 3:**
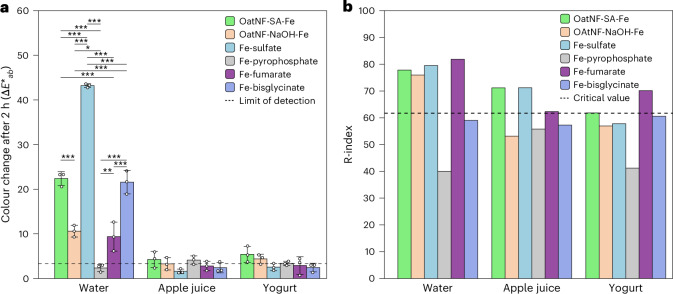
Sensory performance of different iron fortificants in various food matrices. **a**, The colour change impact of OatNF-SA-Fe and OatNF-NaOH-Fe hybrids compared with four commonly used iron fortificants: ferrous sulfate, ferric pyrophosphate, ferrous fumarate and ferrous bisglycinate after their addition to bottled drinking water, apple juice and plain yogurt, 2 h after mixing. *N* = 3 replicates. The data are shown as mean values ± s.d. Data were compared using analysis of variance (ANOVA) for each matrix, with Bonferroni correction applied for multiple comparisons. *P < 0.05, **P < 0.01, ***P < 0.001. **b**, The sensory performance of OatNF-SA-Fe and OatNF-NaOH-Fe hybrids in water, apple juice and yogurt compared with various commercial iron fortificants: ferrous sulfate, ferrous fumarate, ferric pyrophosphate and ferrous bisglycinate. Source data

The changes in colour are shown in Fig. 3a. When added to apple juice or plain yogurt, all compounds induced colour changes that were either below or only slightly above the method’s detection limit. These changes were not visible to the naked eye. In water, by contrast, all compounds except ferric pyrophosphate, a water-insoluble inert compound, produced detectable colour changes. In this matrix, ferrous sulfate induced the most pronounced colour change, followed by OatNF-SA-Fe and ferrous bisglycinate, and then OatNF-NaOH-Fe and ferrous fumarate. The colour changes observed with ferrous sulfate and OatNF-SA-Fe, both soluble forms of iron, are probably due to the oxidation of Fe(II) to Fe(III) at neutral pH in the presence of oxygen, leading to the formation of suspended reddish ferric hydroxide. OatNF-NaOH-Fe produced notably better results than ferrous sulfate, OatNF-SA-Fe and ferrous bisglycinate. The colour changes associated with ferrous fumarate and ferrous bisglycinate can be explained by their intrinsic colour and solubility: ferrous bisglycinate, being highly soluble, yields a dark brown-green solution, whereas the poorly soluble ferrous fumarate results in a light-reddish solution. The absence of visible colour changes in apple juice and yogurt can be attributed to the low pH of these matrices (approximately pH 3 for apple juice and 4-4.5 for yogurt), which protects against oxidation, as well as to masking effects from the inherent colour or opacity of the matrices.

We then moved to investigate sensory performance of the various compounds by a group of trained panellists. To disentangle the colour change effects from the sensory perception, the study was run under red light except for yogurt, thus masking colour changes. We first attempted to quantitatively evaluate metallic taste as a discriminant on a linear scale, but the results did not show significant differences at the level of 2.5 mg of iron, indicating that the sensory impact of all the various compounds at a fortification level of 2.5 mg iron per serving is not significant when colour changes are made undetectable under red light. We then assessed whether changes (positive or negative) were detectable compared with the reference compound using the R-Index method. The number of panellists was 15 for water and 16 for both apple juice and yogurt. Therefore, the number of comparisons in the pooled data was 30 for water and 32 for apple juice and yogurt. Under the null hypothesis (no difference), the expected R-Index is 50 (random guessing) and the threshold was set so that the probability of observing an R-Index this high (or higher) by chance was < 5% (one-sided, *P* < 0.05). Thus, for water, the critical value was 61.9; for apple juice and yogurt, the critical value was 61.6.

The results are shown in Fig. 3b. As expected, the R-Index values for ferrous fumarate and ferrous sulfate were consistently higher than cut-off in all three matrices, with the only exception being ferrous sulfate in yogurt. The R-Index values for ferric pyrophosphate and ferrous bisglycinate were lower in all matrices, with ferrous bisglycinate in yogurt approaching the critical R-Index value for a significant difference. The two study compounds showed intermediate results. The OatNF-SA was sensorially noticeable in water and apple juice and was not clearly differentiated from the reference sample in yogurt. The OatNF-NaOH could sensorially be perceived differently from the reference in water but was well masked in both apple juice and yogurt.

### Clinical iron absorption study in iron-deficient Thai women

We conducted a prospective, cross-over study in young iron-deficient women in Nakhon Pathom, Thailand, a region in Southeast Asia where women are at high risk of iron deficiency anaemia^1^. To measure iron absorption from the different iron compounds, we used a multiple iron stable isotope technique, allowing each participant to serve as her own control. The method is based on the incorporation of isotopic labels into erythrocytes 2 weeks after oral administration^17^.

**Fig. 4:**
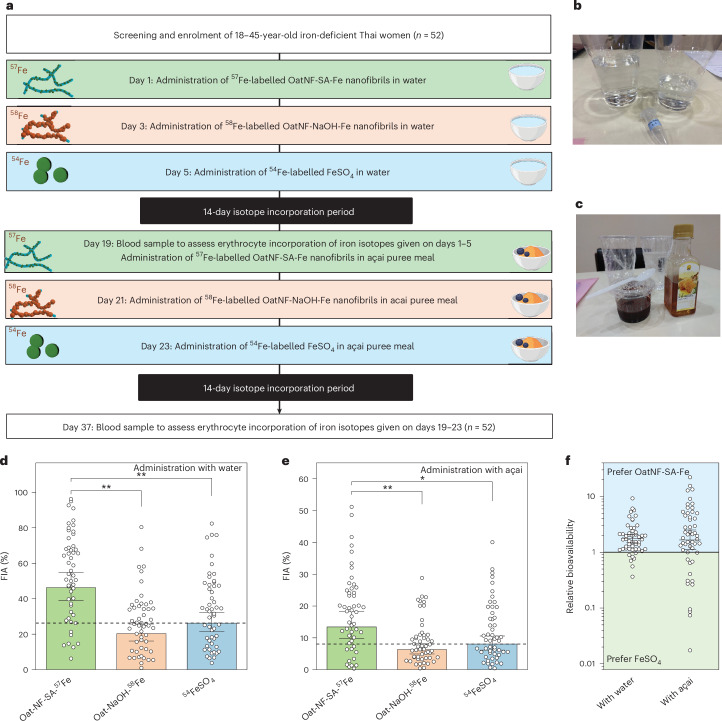
Clinical study to evaluate iron absorption from OatNF-SA-^57^Fe, OatNF-NaOH-^58^Fe and ^54^FeSO_4_ in iron-deficient Thai women. **a**, Outline of the randomized-order, cross-over study design. **b**,**c**, OatNF-SA-Fe compounds dissolved in water (**b**) or mixed in açai puree (**c**) before administration. **d**,**e**, FIA in water from OatNF-SA-Fe, OatNF-NaOH-Fe and FeSO_4_-fortified foods, showing 46.2% (39.1–54.7%), 20.3% (16.1–25.7%) and 26.2% (21.3–32.3%) respectively (**d**), and 13.4% (9.8–18.3%), 6.3% (4.9–8.1%) and 8.1% (6.1–10.6%), respectively, when administered with açai puree (**e**). Data are presented as geometric means with 95% CIs (*n* = 52 participants). Compared using two-sided paired *t*-tests with Bonferroni adjustment for multiple testing. Dashed lines indicate the values of the FeSO_4_ reference. Two absorption values >60% in the first condition are not shown to improve figure clarity. **f**, The relative bioavailability of OatNF-SA-Fe compared with FeSO_4_ was 1.76 (1.48–2.1) when administered with water and 1.65 (1.12–2.51) with açai puree. Data are presented as geometric means ± 95% CIs (*n* = 52 participants) and analysed by one-way ANOVA with Bonferroni correction. *P < 0.05, **P < 0.01. Icons of the food matrix in **a** created with BioRender.com. Source data

As shown in Fig. 4a, the 37-day study was designed so that each participant received six different dietary conditions. The three iron compounds tested were: (1) ^57^Fe-labelled OatNF-SA-Fe hybrid, (2) ^58^Fe-labelled OatNF-NaOH-Fe hybrid and (3) ^54^Fe-labelled ferrous sulfate (FeSO_4_) as the reference compound. Each dose contained 4 mg of elemental iron, and all compounds were intrinsically labelled with stable isotopes during synthesis. The compounds were administered either in water or, to provide a polyphenol-rich inhibitory food matrix, mixed into açai puree with honey (Fig. 4b,c). The primary outcome was fractional iron absorption (FIA), and the prespecified comparisons were both hybrids versus FeSO_4_ in each of the two matrices.

During the screening phase, women aged 18–45 years with serum ferritin (SF) concentrations <50 µg l^−1^ (*n* = 52) were recruited. Detailed inclusion and exclusion criteria are provided in the Methods. The baseline characteristics of the participants are presented in Supplementary Table 1. Eligible participants received the three iron compounds with water on study days 1, 3 and 5. After a 14-day period to allow the iron isotopes to incorporate into erythrocytes, we collected venous blood samples to assess iron absorption from the first three conditions. Participants then received a second round of conditions, with the three iron compounds administered mixed into polyphenol-rich açai puree with honey on study days 19, 21 and 23. The order of the conditions was randomized for each participant for each block using a Python script. The sequences were generated so that each isotope was used only once in each block. Assignment was not masked. After another 14 days, we again collected venous blood samples to measure iron absorption from the final three conditions. In total, 312 measures of iron absorption were conducted across the 52 participants.

Figure 4d shows iron absorption from the three compounds in water. Geometric mean FIA from FeSO_4_ was 26.3% (95% CI 21.4–32.4%). The OatNF-SA-Fe hybrid was remarkably better absorbed than FeSO_4_, exhibiting a high FIA of 46.2% (95% CI 38.9–55.0%). Notably, iron from the OatNF-SA-Fe hybrid was 76% better absorbed (that is, 1.76 times) than iron from FeSO_4_, which is fully water soluble and considered the reference compound for iron absorption. This finding may be due to the very large specific surface area of the subnanometre-sized ferrous iron in the OatNF-SA-Fe hybrid, which enables rapid dissolution at gastric and duodenal pH during digestion, allowing greater uptake by the divalent metal transporter (DMT)-1 (ref. ^18^). In addition, the SA (at a 2:1 molar ratio to iron) probably enhances iron absorption, due to its reducing effect^35^. The OatNF-NaOH-Fe hybrid showed a geometric mean FIA of 20.3% (16.1–25.7%). This corresponds to 77% relative bioavailability compared with FeSO_4_ (that is, 0.77 times) which is considered a moderate-to-high iron bioavailability value for potential iron fortificants^5^. In contrast to the OatNF-SA-Fe hybrid, up to 60% of the iron in the OatNF-NaOH-Fe hybrid is in the ferric form (Supplementary Fig. 3d), and this includes very poorly water-soluble iron hydroxide and iron oxides, which typically exhibit absorption rates of <5% relative to FeSO_4_(ref. ^5^). The high absorption of the subnanometre-sized ferric iron in the OatNF-NaOH-Fe hybrid is also probably due to its very large specific surface area enabling rapid dissolution at low gastric pH, as previously demonstrated for non-hybrid subnanometre-sized iron oxides and phosphates^18^. Finally, the presence of OatNF may have contributed to the high FIA of both hybrids due to the high glutamine content in oat protein, accounting for up to 13% of the amino acid content (Supplementary Fig. 4q). Glutamine has previously been shown to enhance iron absorption in vivo^36^.

Dietary polyphenols strongly inhibit iron absorption by forming unabsorbable complexes with iron in the gut lumen^37,38^. Previous studies have reported reductions in iron absorption from foods and supplements due to polyphenols ranging from 40% to 90% (refs. ^37–41^). Our findings agree with these studies: the polyphenol-rich açai puree meal reduced iron absorption (Fig. 4e) from the three iron compounds by approximately 70% compared with the water condition. However, absorption from the OatNF-SA-Fe hybrids remained high at 13.4% (95% CI 9.8–18.3%), which was 65% higher than absorption from FeSO_4_, which was 8.1% (95% CI 6.1–10.6%). The higher absorption from the OatNF-SA-Fe hybrids may be partly attributed to the presence of SA in the hybrids, as ascorbic acid is known to reduce the inhibitory effects of polyphenols on iron absorption^42^. This suggests that the OatNF-SA-Fe hybrid remains highly bioavailable even when incorporated into polyphenol-rich foods, underscoring its potential as an effective iron fortificant. Figure 4f and Supplementary Fig. 5 show the relative bioavailability of OatNF-SA-Fe, OatNF-NaOH-Fe and FeSO_4_ when administered with water and with açai puree, normalized for each individual participant.

## Discussion

We introduce in this work a previously unexplored protein–iron nanoparticle hybrid system based on OatNF carrying ultrasmall iron nanoparticles. The synthesis can be tailored to produce either ferrous or ferric iron nanoparticles, depending on the choice of reducing agent used. When using SA, OatNF carries stabilized ferrous iron that is remarkably well absorbed in humans, with a mean fractional absorption of 46% in water—76% higher bioavailability compared with FeSO_4_, the reference compound. When using NaOH, OatNF carry mainly ferric iron, which is still well absorbed and shows superior sensory performance in reactive food matrices. Based on these results, we interpret the main role of oat nanofibrils to be the stabilization of Fe(II) in the formulation. The OatNF-NaOH-Fe control is particularly informative: by renormalizing the clinical iron absorption in Fig. 4a to the total Fe(II) present at the start, we find absorption efficiencies of 26.3% (^54^Fe), ~51% (^57^Fe) and ~58% (^58^Fe). This clearly demonstrates that protein nanofibrils effectively preserve Fe(II), independent of its starting concentration. By contrast, the primary function of SA appears to be enhancing the initial Fe(II) content, rather than affecting its preservation or absorption directly. Nonetheless, both ascorbate reduction of ferric iron in the gut lumen and increased enterocyte ascorbate^43^ may further contribute to the high bioavailability observed for OatNF-SA-Fe.

OatNF exhibit several key advantages as an iron delivery platform for human nutrition: (1) This oat-based formulation is suitable for all populations, even those with plant-based diets, which typically have both inadequate iron intake and low bioavailability^44,45^. (2) OatNF provide efficient binding sites for subnanometre iron particles. This binding and alignment is probably due to the intrinsic protein affinity to form supramolecular complexes^46,47^. Our results clearly demonstrate that the exceptional bioavailability of iron observed in the OatNF-SA diet is due to the role of protein nanofibrils. Indeed, as evident from the analysis in both solid and dispersion by XPS and colorimetric assays, respectively (Fig. 2 and Supplementary Figs. 4i,n), the initial amount of Fe(II) in the oat hybrids and iron sulfate is essentially identical. Therefore, the superior iron bioavailability observed for OatNF-SA hybrids can only be attributed to the differing fate of Fe(II) during digestion and absorption. (3) OatNF contain abundant reducing amino acids^20^ and a high content of antioxidant peptides^48,49^ that are capable of stabilizing subnanometre ferrous iron nanoparticles in the OatNF-SA-Fe hybrids even during long storage (Fig. 2h). (4) the presence of OatNF nanofibrils maintains iron in a colloidally stable state, preventing aggregation and preserving bioavailability. (5) Because of their high solubility in water, acceptable organoleptic sensory features and pleasant oat flavour and aroma, OatNF-SA-Fe hybrids may be efficient iron fortificants for a wide variety of beverage and food matrices.

The digestion and absorptive pathway of OatNF hybrids remains uncertain. Based on our INFOGEST experiment results, both oat nanofibril formulations underwent substantial degradation during gastric digestion due to pepsin hydrolysis, followed by complete digestion during the intestinal phase. We thus hypothesize that the iron released from the digested protein fibrils—whether in ferric or ferrous form—primarily enters the common non-haem iron pool in the stomach and duodenum. Ferric iron is then probably reduced by duodenal cytochrome b (DcytB), a ferric reductase located on the apical surface of enterocytes, or by ascorbic acid in the case of the OatNF-SA-Fe formulation^6^. The resulting ferrous iron is probably absorbed through the conventional divalent metal transporter 1 (DMT1) pathway, along with any iron already present in the ferrous form. Given the complete digestion and dissolution observed in the INFOGEST experiment, and consistent with previous work by Shen et al.^20^, it is unlikely that intact OatNF hybrids are taken up via endocytosis, although we cannot entirely exclude the possibility that trace amounts may be absorbed this way. It is also possible—although less likely—that a small portion of iron remains bound to amino acids or peptide fragments after digestion rather than fully solubilizing. These iron–amino acid complexes, similar to aminochelated iron, could be absorbed through an alternative pathway^50^. However, this pathway remains speculative. For example, a study using DMT1-knockout Caco-2 cells^51^ found no iron uptake from ferrous bisglycinate, casting doubt on the physiological relevance of this mechanism.

In summary, we developed a food-grade oat protein nanofibril-based iron compound for beverage and food fortification that delivers iron with exceptionally high bioavailability. This study demonstrates the potential of protein–iron nanoparticle hybrid systems for effective iron absorption in humans. In iron-deficient women, a key target group for iron fortification, the OatNF-SA-Fe hybrid demonstrated exceptional FIA. The geometric mean absorption was 46.2% when administered with water and 13.4% when given with a polyphenol-rich food matrix, achieving very high values of bioavailability corresponding to 176% and 165% those of FeSO_4_. Moreover, the OatNF hybrids exhibited sensory performance equal to or better than FeSO_4_ in fortified foods and beverages. These results make OatNF hybrids an innovative and highly effective approach to iron delivery in nutritional applications, which could contribute to reducing the global burden of iron deficiency and anaemia.

## Methods

### Materials

The following materials were used: oat flour (Hafermehl grob, SWISSMILL), *n*-hexane (LiChrosolv, CAS-No: 110-54-3, Merck KGaA), NaOH (BioXtra, ≥98%, pellets, CAS-No: 1310-73-2, Sigma-Aldrich), NaCl (≥99.5%, CAS-No: 7647-14-5, Sigma-Aldrich), extracted oat protein, HCl (1.09057, CAS-No: 7647-01-0, Sigma-Aldrich, Reagent European Pharmacopoeia Standard), FeCl_3_, SA (Sigma-Aldrich 134-03-2; Reagent European Pharmacopoeia Standard), NaOH (1.09137, CAS-No: 1310-73-2, Sigma-Aldrich, Reagent European Pharmacopoeia Standard), hydrogen peroxide 30% (1.07298, CAS-No: 7722-84-1, Sigma-Aldrich, Suprapur) and ^54^Fe-, ^57^Fe- and ^58^Fe-enriched elemental iron powders (Chemgas). All reagents were of European Pharmacopoeia reagent grade. All steps were carried out with food-grade equipment in the pilot plant/food laboratory under food-grade conditions.

### Oat protein extraction and fibrillization

The oat protein extraction followed our previous protocol^22^. We also improved the protocol to enhance the yield while maintaining the same nanofibril quality. In brief, oat flour was defatted with *n*-hexane (1:3 w/v) three times for 1 h, centrifuged at 5,000*g* for 15 min and air-dried for 24 h. The defatted powder was then dispersed in 1 M NaCl pH 10 (1:10 w/v) and gently stirred for 2 h at 20 °C before centrifugation (7,000*g*, 15 min, 20 °C). The protein-rich supernatant was diluted with Milli-Q water (1:6.66 v/v) and left 12 h at 4 °C without agitation to precipitate the dissolved OGs. After centrifugation (7,000*g*, 15 min, 20 °C), the pellet was resuspended in Milli-Q water and dialysed (molecular weight cut-off: 6–8 kDa, Spectra/Por RC membrane, Spectrum Laboratories) against Milli-Q water for 24 h to remove the salt. The dialysed sample was collected, frozen and subsequently freeze-dried to obtain the OG powder.

To fabricate OG nanofibril, extracted OG was first dissolved in Milli-Q water (pH 2) at 2 wt%. After stirring 5 min in ambient conditions, the solution pH was readjusted to pH 2. The dissolved protein solution was then incubated at 90 °C in an oil/water bath for 18 h while stirring at 350 rpm.

### Production of OatNF-SA-Fe and OatNF-NaOH-Fe hybrids

For OatNF-SA-Fe hybrid, the freshly prepared OG nanofibril solution (2 wt%) was first mixed with MQ water and FeCl_3_ solution (0.3 M). Then, the freshly dissolved SA (3 M) was added drop by drop, followed by a gentle mixing of the dispersion. The final dispersion reached FeCl_3_ at 100 mM and SA at 200 mM. For samples for clinical studies, the iron concentration was determined by atomic absorption spectroscopy. Dispersion with 4 mg iron was loaded in each Eppendorf and freeze-dried. The Eppendorf was then filled with N_2_, which was subsequently replaced with argon before shipping for clinical studies.

For the OatNF-NaOH-Fe hybrid, the freshly prepared OG nanofibril solution (2 wt%) was first mixed with MQ water and FeCl_3_ solution (0.3 M) and vortexed for 30 s. The dispersion was then carefully adjusted to pH 7 by adding NaOH (7.5 M) droplet by droplet. The final dispersion contained FeCl_3_ at 100 mM. For samples for clinical studies, the iron concentration was determined by atomic absorption spectroscopy. Dispersion with 4 mg iron was loaded in each Eppendorf, frozen and stored with dry ice before shipping for clinical studies.

In clinical study, FeCl_3_ solutions were prepared by dissolution of ^57^Fe and ^58^Fe elemental powders in stochiometric amounts of 6 M HCl, followed by oxidation of Fe(II) to Fe(III) with equimolar amounts of 30% hydrogen peroxide.

### STEM

For STEM imaging and analytical analyses, an aliquot of samples (5 μl) was deposited on 2-nm carbon-coated lacey grids (Quantifoil, D) pretreated by glow discharge (Pelco easyGlow) for 2 min to ensure the optimal material distribution. The excess fluid was removed with filter paper, and the grids were washed three times with distilled water and air-dried.

The ambient temperature STEM measurements were performed using TFS Talos F200X (Thermo Fisher Scientific) and JEOL JEM-F200 (JEOL) instruments both operated at 200 kV and a double *C*_s_-corrected JEOL JEM-ARM300F GrandARM ‘Vortex’ (JEOL) instrument operated at 300 kV. All instruments equipped with cold-emission FEG sources were used in a scanning TEM mode. For imaging, the HAADF and circular bright-field detectors were used on all three instruments. A STEM probe with a diameter of approximately 0.25 nm was used (condenser aperture 70 μm, convergence angle 10.5 mrad). STEM illumination and acquisition parameters were chosen such that the low-angle annular dark field STEM and circular bright-field STEM detectors yielded the diffraction contrast information, whereas the HAADF-STEM detector provided the prominent atomic number contrast from the same probe position.

### EDS

The materials elemental content and its distribution in the specimens were assessed by EDS measurements using TFS Talos F200X (Thermo Fisher Scientific, USA) equipped with the SuperX EDS module using the Esprit I package for the evaluation and analyses of spectrum images. The corresponding STEM probe size was set to about 0.25 nm (convergence angle 10.5 mrad, condenser aperture 70 mm), providing the high-resolution imaging and simultaneously yielding sufficient electron probe current for high X-ray count rates. The EDS STEM elemental maps were produced and evaluated using the Esprit I software of the module.

### EELS

The hybridization states of Fe in the compounds were assessed using EELS modules of both JEOL instruments GrandARM (JEOL) and JEM-F200 (JEOL). The GIF Quantum ER EELS spectrometer on the GrandARM and the Gatan Continuum S EELS spectrometer on the JEM-F200 enabled acquisition of EEL spectra in dual EELS mode. The studies were carried out using 5–7 mA of the emission current and energy dispersion of 0.15 eV per channel to include both, O-K and Fe-L_23_ edges in the spectra.

### Cryo-TEM

Cryo-TEM samples were prepared in a Vitrobot Mark IV (Thermo Fisher Scientific) at 22 °C and 100 % humidity. Three microlitres of sample were added on hydrophylized lacey carbon-coated copper grids (EMS), and the grids were plunge frozen into a mixture of liquid ethane and propane cooled by liquid nitrogen. The vitrified grids were clipped into AutoGrid sample carriers (Thermo Fisher Scientific) for automated loading.

The sample morphology was verified using cryo-TEM to assess potential electron-beam-induced material damage. The data acquisition was carried out on a TFS Titan Krios (Thermo Fisher Scientific) operated at 300 kV of acceleration voltage and equipped with a Gatan Quantum-LS Energy Filter (GIF) and a Gatan K2 Summit direct electron detector (Ametek Pleasanton). The imaging of the vitrified materials on a cryo-stage constantly kept at 80 K was performed in an energy-filtered TEM (EFTEM) operation mode using the TFS EPU software and K2 camera in a linear mode.

### XPS

XPS measurement was performed in a PHI Quantera system. Dried samples were pressed into In foil before measurement and measured at a pressure of 10^−9^–10^−8^ torr. The monochromatic Al Kα radiation was generated from an electron beam (24.8 W) with a 100 μm spot size. Charge neutralization was performed using a low-energy electron source. Peak fitting of photoelectron features was performed in Casa XPS using Voigt profiles with GL ratios of 40 following Tougaard background subtraction for the Fe 2*p* region and Shirley background subtraction for all other cases. For Fe 2*p* fitting, we used the approach described by A. P. Grosvenor et al. to constrain the relative peak positions^25^. Relative peak areas between main and satellite peaks were fixed between each analysed Fe 2*p*spectrum. Furthermore, the full width at half maximum was fixed to be identical between each main peak and its corresponding satellite peak. Binding energy was referenced to the single N 1*s* feature located at 499.7 eV, based on literature values for amide bonds^52^, using this peak as the internal charge reference.

### Colour change measurements

Colour change measurements were performed by adding amounts of ferrous sulfate heptahydrate, ferric pyrophosphate, ferrous fumarate, ferrous bisglycinate (all food grade, obtained from Dr. Paul Lohmann, Emmerthal, Germany), OatNF-SA-Fe hybrids and OatNF-NaOH-Fe hybrids, containing 2.5 mg of Fe to either 100 ml of bottled water and apple juice or 100 g of plain yogurt 1.1% fat. All products were from the M-Budget line and were purchased at the Migros supermarket chain, Zurich, Switzerland. All fortified beverages and foods were prepared in triplicate.

Colour change was measured after 2 h of standing at room temperature and compared with beverages or foods without added iron compounds. Absolute colour change (ΔE^*^_ab_) was determined using a spectral photometer (Chroma Meter CR-410, Konica-Minolta) in the Hunter Lab colour system, calculated as follows:

$$\Delta {E}_{{ab}}^{* }=\,\sqrt{{\left(\Delta {L}^{* }\right)}^{2}+{\left(\Delta {a}^{* }\right)}^{2}+{\left(\Delta {b}^{2}\right)}^{2}},$$

where ΔL* (lightness), Δa* and Δb* (chromaticity coordinates) correspond to the difference between the sample (with added compound) and the not fortified matrix.

The SPSS statistical programming environment (IBM SPSS Software, Version 28) and Microsoft Office EXCEL 2016 (Microsoft) were used for the data analysis. Comparisons were done using two-sided paired *t*-tests with Bonferroni adjustment for multiple testing. *P* values <0.05 were considered statistically significant.

### Sensory study

#### Participants

The participants were recruited from among the staff and students of the Department of Health Sciences and Technology at ETH Zurich in Switzerland. Sixteen participants, with an average age of 25.9 years (median age 24), volunteered to participate in the study. By signing a consent form, they confirmed that they had no food allergies or intolerances and would follow the tasting protocol during all sessions. The study was approved by the ETH Zurich ethics committee (25 ETHICS-166).

#### Sample preparation and presentation

All samples were prepared hygienically on the morning of the tasting sessions. Commercial samples of bottled still water, cow’s milk yogurt 1.1% fat and regular apple juice, all from the M-Budget line (Migros supermarket, Zurich, Switzerland), were used as matrices to test the sensory impact of different iron compounds. Four food-grade commercial iron compounds (ferrous sulfate, ferric pyrophosphate, ferrous bisglycinate and ferrous fumarate, all obtained from Dr. Paul Lohmann, Emmerthal, Germany) and the two comparators (OatNF-SA-Fe and OatNF-NaOH-Fe) were mixed at a concentration of 2.5 mg Fe per 100 g in a 100-g matrix. Two centilitres of the mixed samples were transferred to 4-cl cups. Ferrous fumarate and ferric pyrophosphate were insoluble in water and apple juice; OatNF-NaOH-Fe was only partially soluble. Therefore, all liquids had to be stirred by the panellists before tasting and were served at room temperature under red light to avoid colour bias. The yogurt samples were stored at 4 °C until the tasting session and were served with small spoons. All cups were labelled with three-digit codes, and the samples were presented to the panellists in a balanced design. The testing room temperature was about 22 °C.

#### Sensory evaluation

All participants attended a training session. This included an introduction to the tasting protocol and familiarization with different matrices containing a selection of iron compounds, as well as the evaluation methods. Iron sulfate was considered a standard compound because it is used as a reference in sensory training (ISO 8586 (ref. ^53^)). Panellists were instructed to take a sip, evaluate the sample and spit it out again, followed by neutralizing their palate with water and crackers. Liquid samples had to be stirred. The yogurt samples were served with small spoons. The training block included a descriptive task to get a consensus on the attribute metallic as well as a ranking test. The panellists ranked four samples with increasing concentrations of iron sulfate in water (1, 2.5, 5 and 10 mg per 100 ml water), using the attribute ‘metallic’. The panellists were given feedback about their performance and were allowed to retaste the samples. The same concentrations were rated on a 100-unit line scale with respect to the metallic attribute, including selected compounds. For data collection, the concentration was restricted to 2.5 mg Fe per 100 g of matrix. Based on the pretests and training results, multiple difference testing was decided upon, as differences with only 2.5 mg of iron were found to be minimal and difficult to be rated on line scales. All six compounds were tested in their respective matrices and presented in blocks of seven samples. The bland matrix was presented as a reference. Panellists had to evaluate whether a sample was the same as the reference and whether they were confident in their decision (four levels: R sure, R unsure, not R unsure and not R sure). The panellists tested all samples in duplicate, including the reference, for a total of 14 samples per matrix. The tastings were conducted on two consecutive days. Results were pooled, and the R-Index was calculated^54^. The tables of Bi and O’Mahony^55^ were used to test for significance (*P* = 0.05, one-sided).

### Human study

The study was a single-centre, prospective cross-over stable isotope trial, conducted at the Institute of Nutrition of Mahidol University, in Nakhon Pathom, Thailand. The study protocol is available from the Mahidol University Central Institutional Review Board (MU-CIRB), the study investigators, and online at ClinicalTrials.gov (ID no. NCT05826899). All participants provided written informed consent before any study procedure took place.

#### Participants

The study was conducted in Thailand, and the female participants (*n* = 52) were recruited among students and staff of Mahidol University in the Bangkok metropolitan area. To minimize biological and environmental confounding of FIA, we enrolled non-pregnant, non-lactating women aged 18–45 years with SF <50 µg l^−1^ and haemoglobin (Hb) ≥12 g dl^−1^, and excluded participants with inflammation (C-reactive protein (CRP) >5 mg l^−1^), thalassaemia, recent blood loss/transfusion, chronic disease, tobacco use, recent antibiotic or supplement use, and chronic medications (except oral contraceptives). Body mass index and weight limits were applied to reduce interindividual variability in iron kinetics and dosing per body mass. These eligibility criteria were chosen to strengthen internal validity by removing known modifiers of iron absorption. Data on ethnicity and socio-economic status were not collected. Although recruitment from a university population may limit generalizability to other Thai women, it is unlikely to have introduced systematic bias in the physiological measurement of iron absorption. The recruitment inclusion criteria were as follows: (1) female; (2) age 18–45 years; (3) SF <50 µg l^−1^; (4) body mass index 18.5–24.9 kg m^−^^2^; (5) weight <70 kg; (6) signed informed consent. Exclusion criteria were as follows: (1) anaemia (defined as Hb <12 g dl^−1^); (2) presence of thalassaemia; (3) inflammation (defined as CRP >5 mg l^−1^); (4) cigarette smoking; (5) chronic digestive, renal and/or metabolic disease; (6) chronic medications (except for oral contraceptives); (7) use of vitamin, mineral and pre- and/or probiotic supplements in the previous 2 weeks; (8) blood transfusion, blood donation or important blood loss in the previous 4 months; (9) history of difficulties with blood sampling; (10) antibiotic treatment in the previous 4 weeks; (11) pregnancy (tested in serum at screening); (12) lactation in the previous 6 weeks; (13) prior participation in a study using stable isotopes or participation in any clinical study in the previous 30 days; (14) unable to comply with study protocol.

#### Study procedures

During screening, about 2 weeks before the start of the study, we collected a venipuncture sample (6 ml) for the determination of Hb, SF, CRP, thalassaemia and pregnancy. Body weight and height were recorded, and an interview was conducted to assess inclusion and exclusion criteria. Eligible women were invited to participate in the study.

Each participant received 3 intrinsically labelled Fe compounds, each administered twice in two study phases: (1) 4 mg Fe as OatNF-SA-^57^Fe hybrid, (2) 4 mg Fe as OatNF-NaOH-^58^Fe hybrid and (3) 4 mg Fe as ^54^FeSO4. During the first phase, we administered the compounds on days 1, 3 and 5, diluted in 100 ml reverse osmosis purified water, along with 240 ml purified water as a drink. During the second phase of the study (days 19, 21 and 23), the same labelled compounds were readministered with identical amounts of water, accompanied with 30 g açai puree (The Rainforest Company) sweetened with 5 g honey (Doi Kham brand by Royal Doi Kham Food Products). The order of administration of each compound was randomized for each phase of the study using a Python code. The administrations took place between 7:00 and 9:00 after an overnight fast.

We collected venous blood samples (6 ml) on days 1 (baseline), 19 (phase 1) and 37 (phase 2) to determine Hb, SF, CRP and erythrocyte isotopic composition. Body weight was measured on the same days.

#### Laboratory analysis

Hb, SF and CRP concentrations were measured at the National Healthcare Systems (NHealth) in Bangkok, Thailand, using an automated haematology analyser (Sysmex), a chemiluminescent microparticle immunoassay and immunoturbidimetry, respectively. Anaemia was defined as Hb <120 g l^−1^. Iron deficiency was defined as SF <30 μg l^−1^, and inflammation was described as CRP >5 mg l^−1^. Whole blood aliquots were sent on dry ice to ETH Zurich, where their iron isotopic composition was measured in duplicate by multi-collector inductively coupled plasma mass spectrometry^56^ (Neptune, Thermo Fisher Scientific).

#### Sample size

We based our power calculation on data from our previous iron absorption study that administered submicrometre-sized ferric phosphate to young women in Thailand^19^. In that study, the standard deviation observed between the differences of the logs of the FIA was 0.223. To resolve a difference of 30% in FIA with a power of 80% and a 5% error rate, we estimated the sample size to be 44 participants. To account for a 20% participant drop-out, we recruited 52 participants.

#### Calculations

FIA from the iron supplements was calculated based on the shift of the iron isotopic ratios in the collected whole blood samples, using the principles of isotopic dilution and assuming 80% incorporation of the absorbed iron into the erythrocytes^17^. Circulating iron in the body was estimated based on blood volume, derived from body height and weight, and Hb concentration^17^.

#### Statistical analysis

The SPSS statistical programming environment (IBM SPSS Software, Version 28) and Microsoft Office EXCEL 2016 (Microsoft) were used for data analysis. Data were examined for normality by use of the Shapiro–Wilk test. Normally distributed data were reported as the mean (s.d.), and non-normally distributed data were reported as the median (interquartile range). Comparisons were done using two-sided paired *t*-tests with Bonferroni adjustment for multiple testing. *P* values <0.05 were considered statistically significant.

### Ethics statement

The protocol of the clinical study was approved by the Ethical Board of Mahidol University and was registered at clinicaltrials.gov (ID no. NCT05826899). All participants provided written informed consent before any study procedure took place. Mahidol University Central Institutional Review Board (MU-CIRB) is in full compliance with International Guidelines for Human Research Protection such as the Declaration of Helsinki, The Belmont Report, CIOMS Guidelines and the International Conference on Harmonization in Good Clinical Practice (ICH-GCP. Certificate No. COA No. MU-CIRB 2°023/024.2802). The protocol for the sensory study was approved by the ETH Ethics Commission (no. 25ETHICS-166) and the Zurich Cantonal Ethical Commission (Req-2025-00599).

### Reporting summary

Further information on research design is available in the Nature Portfolio Reporting Summary linked to this article.

## Supplementary information

Supplementary Information Supplementary Figs. 1–6, Table 1, Methods and References.

Reporting Summary

Supplementary Data 1 Source data for Supplementary Figs. 2–4 and 6.

## Source data

Source Data Fig. 2 Statistical source data.

Source Data Fig. 3 Statistical source data.

Source Data Fig. 4 Statistical source data.

## Data Availability

The data in this study are available within the Article and its Supplementary Information. All other data are available upon request to the corresponding authors. Source data are provided with this paper.
