# Supplementary material for: Oat protein nanofibril–iron hybrids offer a stable, high-absorption iron delivery platform for iron fortification
Source: Nat Food. 2025 Nov 10;6(12):1164–75. doi: 10.1038/s43016-025-01260-6 (PMC12717008; doi:10.1038/s43016-025-01260-6)
Supplement: Supplementary file 1 — Supplementary Figs. 1–6, Table 1, Methods and References. [file 43016_2025_1260_MOESM1_ESM.pdf]

nature food

Supplementary information

https://doi.org/10.1038/s43016-025-01260-6

Oat protein nanofibril–iron hybrids offer a stable, high-absorption iron delivery platform for iron fortification

In the format provided by the authors and unedited

Table of Contents

Supplementary Figure 1 2
Supplementary Figure 2 3
Supplementary Figure 3 4
Supplementary Figure 4 5
Supplementary Figure 5 6
Supplementary Figure 6 7
Supplementary Table 1. 8
Supplementary Methods 9
Supplementary References 10

Supplementary Figure 1

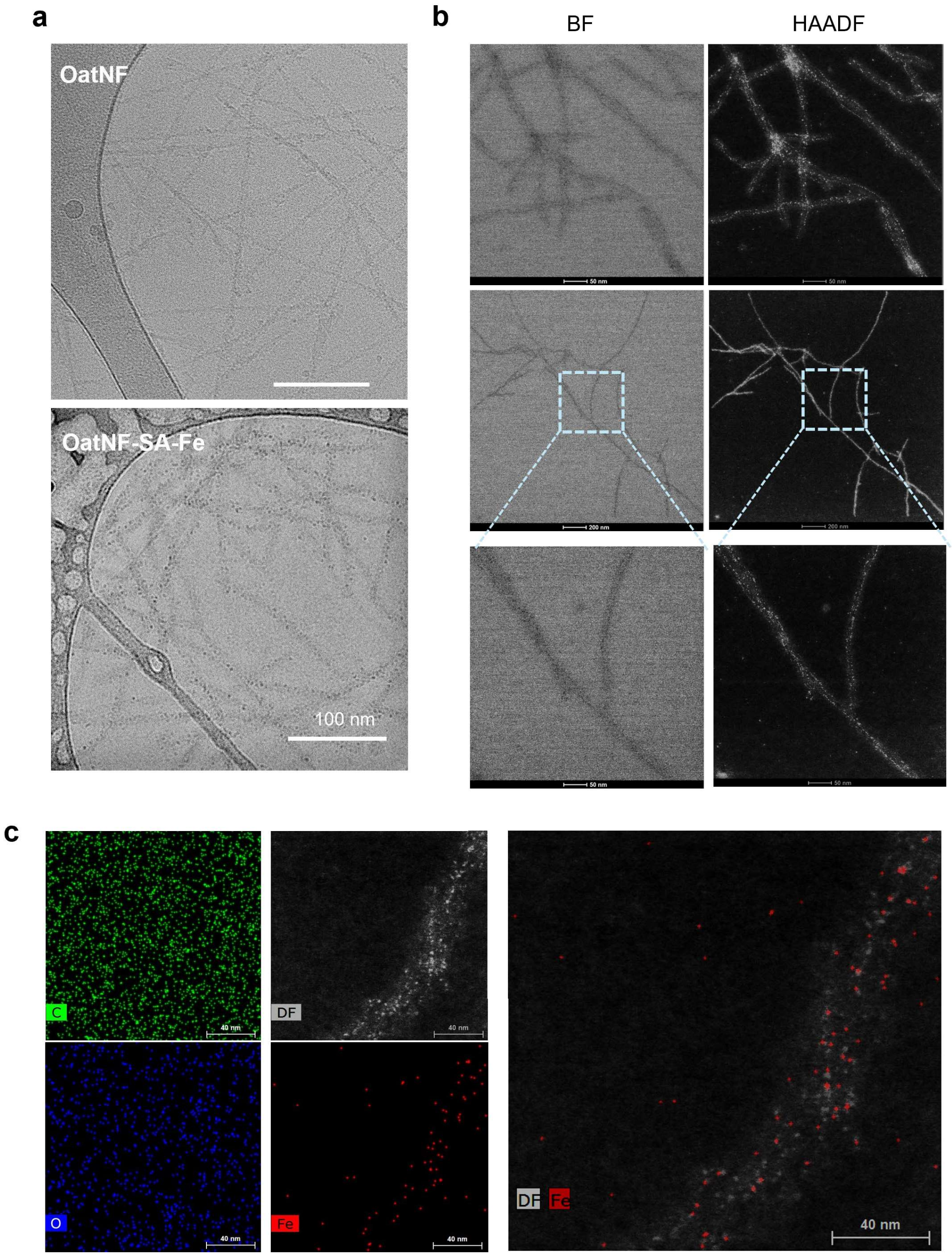

Supplementary Figure 1 | Electron microscopy characterization of synthesized iron fortification OatNF-SA-Fe hybrid. a, CryoTEM images of oat nanofibril before and after reducing iron nanoparticles. **b**, STEM images acquired in the high angle annular dark field (HAADF) and circular bright field (BF) detectors. The former provided the prominent atomic number contrast, and the latter yielded the diffraction contrast information. **c**. EDS elemental mapping images of OatNF-SA-Fe hybrids including C, O and Fe, and the corresponding HAADF-STEM image at the same position. The overlay of HAADF-STEM image and EDS elemental map of Fe indicate the accumulation of iron on the surface of oat nanofibril.

Supplementary Figure 2

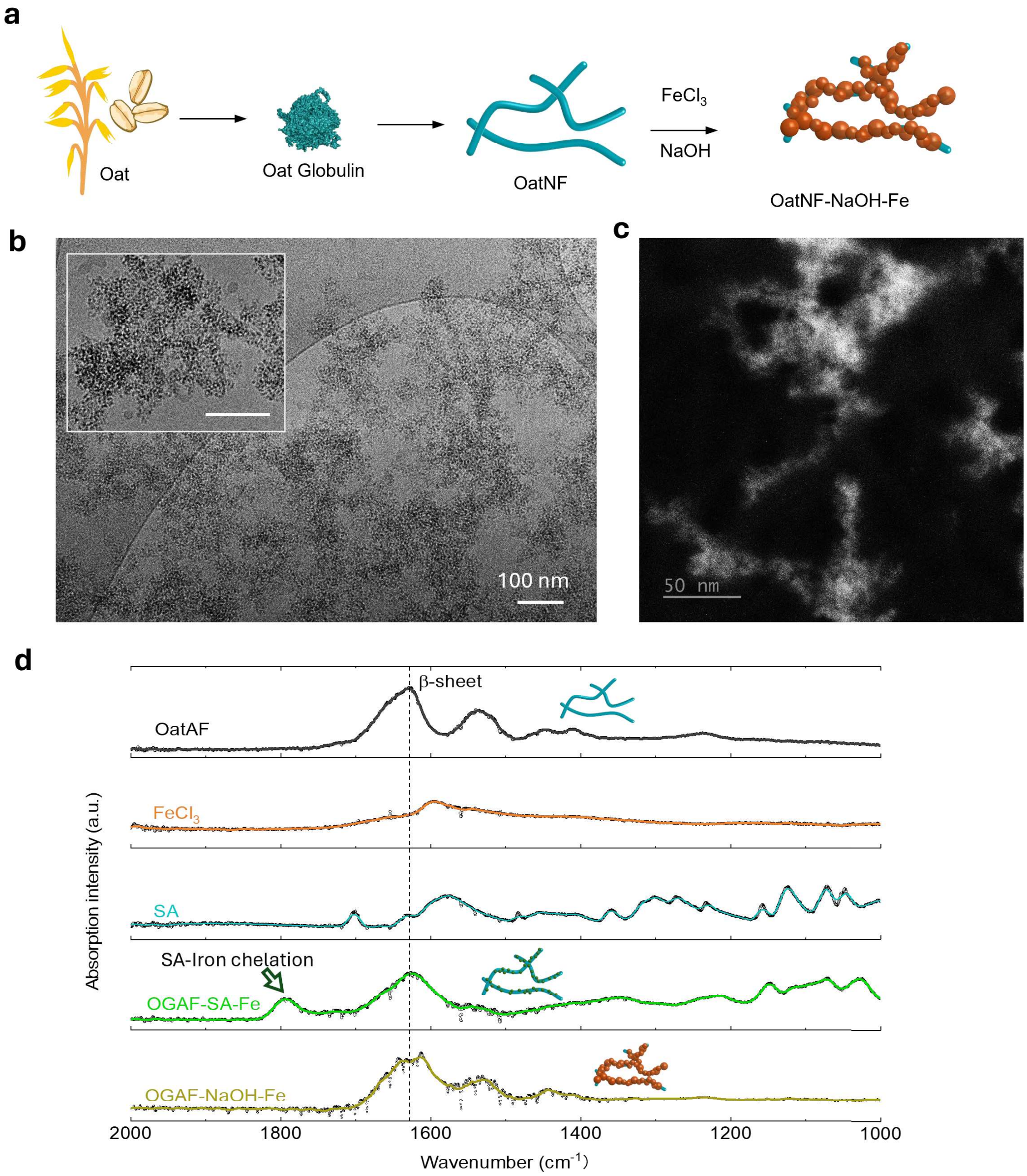

Supplementary Figure 2 | Fabrication of OatNF-NaOH-Fe hybrids and FTIR analysis. a, The illustration of fabrication process of OatNF-NaOH-Fe hybrid. **b**, CryoEM images of oat nanofibril of OatNF-NaOH-Fe hybrid **c**. HAADF-STEM image of OatNF-SA-Fe hybrid, where the bright dots indicate the heavier elements that are iron in the hybrid. The rich iron distribution of oat nanofibril is found in this OatNF-NaOH-Fe hybrid, in line with our previous report[1]. (**d**) FTIR analysis of OatNF, FeCl3, SA, OatNF-SA-Fe and OatNF-NaOH-Fe hybrid. The infrared absorption peak of β-sheet (1625 cm^{-1}) in the Amide I of oat nanofibril is noted, which are found in both OatNF-SA-Fe and OatNF-NaOH-Fe hybrid. The absorption peak at 1795 cm^{-1} in the OatNF-SA-Fe spectrum denotes the SA-iron chelation, which disappears in the OatNF-NaOH-Fe spectrum. This indicates the effect of SA in preventing iron aggregation and promoting sub-nm iron-binding on the OatNF surface.

Supplementary Figure 3

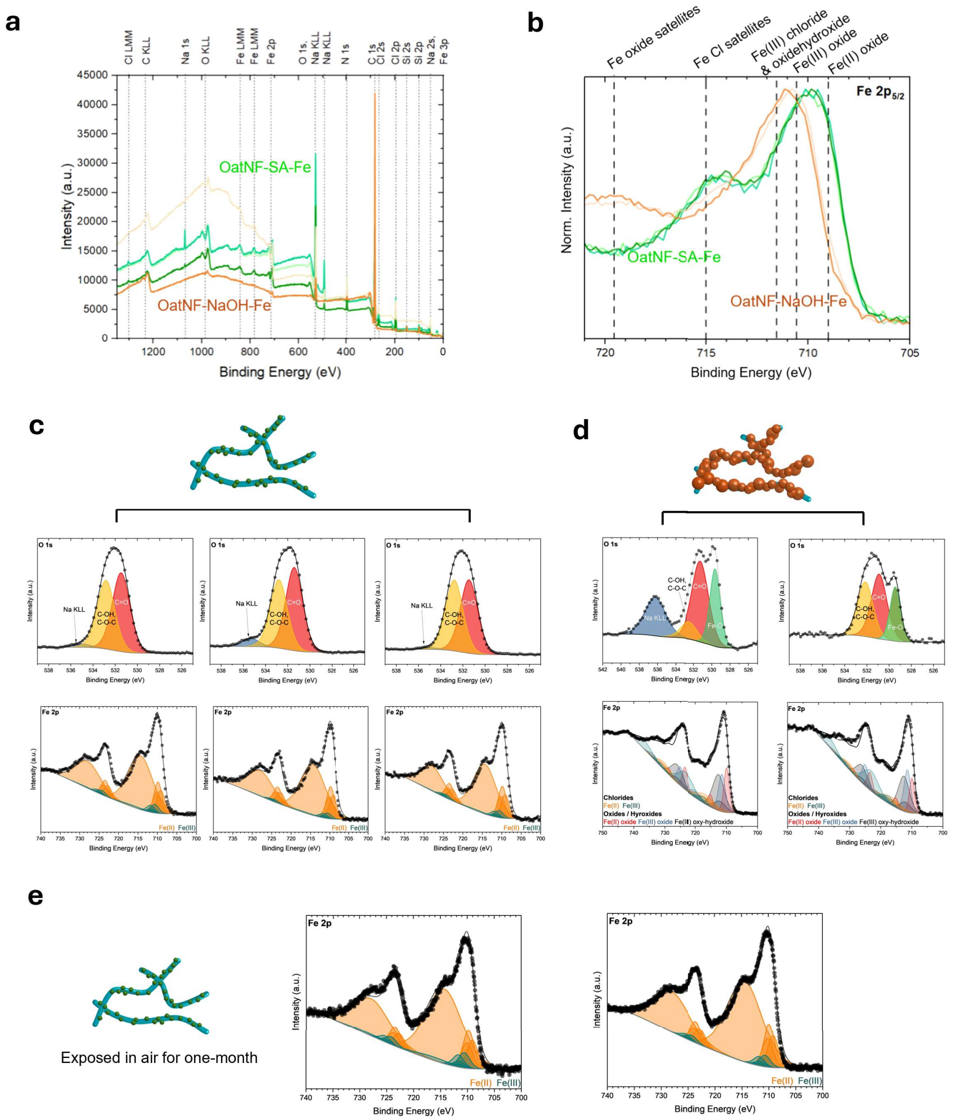

Supplementary Figure 3 | XPS characterization of OatNF-SA-Fe and OatNF-NaOH-Fe hybrids. a-b, XPS Survey spectra (**a**) and XPS Fe $2p_{5/2}$ region (**b**) of OatNF-SA-Fe and OatNF-NaOH-Fe hybrids. The depicted reference lines are based on a previous investigation of the pure compounds by A.P. Grosvernor *et al.*[2]. **c**, Fitted XPS O 1s core level (upper) and fitted XPS Fe 2p core level regions (lower) of OatNF-SA-Fe hybrid, and the. There is no clear Fe-O bond found in the hybrid and the fitting based on literature[2] showed approximate (92±5)% Fe(II) in the OatNF-SA-Fe hybrid (Fig. 2h). **d**, Fitted XPS O 1s core level (upper) and fitted XPS Fe 2p core level regions (lower) of OatNF-NaOH-Fe hybrids. Clear Fe-O bonds showed in the O 1s spectra indicating the composition of iron oxides and iron oxy-hydroxide. The fitted Fe 2p spectra confirmed these composition and indicated approximately 30-40% Fe(II) species in the OatNF-NaOH-Fe hybrid. **e**, XPS analysis on the OatNF-SA-Fe hybrid after one-month exposure in air. The similar spectra were detected and fitted results revealed (88±5)% and (93±5)% Fe(II) content in the OatNF-SA-Fe hybrid. While these results provide a valuable comparison between samples, they are semi-quantitative in nature.

Supplementary Figure 4

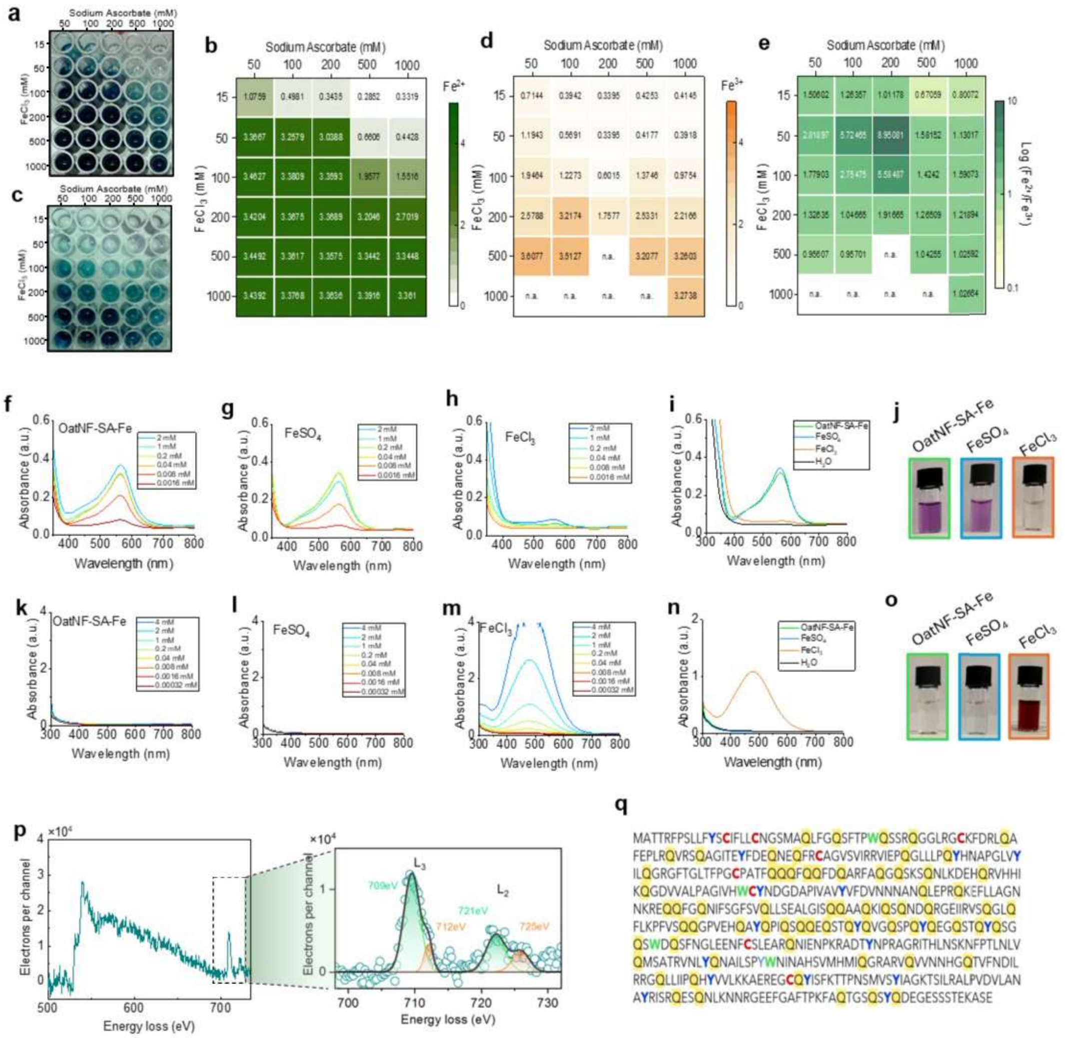

Supplementary Figure 4 | Characterization of OatNF-SA-Fe hybrids. a-b, The determination of Fe(II) through potassium ferricyanide colorimetric assay. The photo (**a**) and the absorbance (**b**) using a spectrophotometer of OatNF-SA-Fe dispersion at different SA and $FeCl_3$ concentrations were indicated. **c-d**, The determination of Fe(III) through colorimetric assay by potassium ferrocyanide reagent. The photo (**c**) and the absorbance (**d**) using a spectrophotometer of OatNF-SA-Fe dispersion at different SA and FeCl3 concentrations were indicated. **e**, The determination of Fe^{2+}/Fe^{3+} ratio through the colorimetric assay by potassium ferricyanide and potassium ferrocyanide reagent. **f-j**, The determination of Fe(II) through colorimetric assay by ferrozine in the OatNF-SA-Fe dispersion (**f**), $FeSO_4$ solution (**g**) and $FeCl_3$ solution (**h**) at different concentration. Their comparison at Fe concentration of 1mM (**i-j**) indicates identical, high Fe(II) content in the OatNF-SA-Fe dispersion and $FeSO_4$ solution. **k-o**, The determination of Fe(III) through ammonium thiocyanate colorimetric assay in the OatNF-SA-Fe dispersion (**k**), $FeSO_4$ solution (**l**) and $FeCl_3$ solution (**m**) at different concentrations. Their comparison at Fe concentration of 1 mM (n-o) indicates low Fe(III) content in the OatNF-SA-Fe dispersion and $FeSO_4$ solution. **p**, EELS spectra of our OatNF-SA-Fe hybrids. **q**, The protein sequence of oat globulin[3]. The amino acids (AAs) with reducing and antioxidant activity[4] including cysteine, tryptophan and tyrosine are marked, that contribute to the reduction of ferrous iron on the surface of OatNF. The glutamine AA is also highlighted in the protein sequence, account for up to 13% of total AAs, which might promote *in vivo* iron uptake.

Supplementary Figure 5

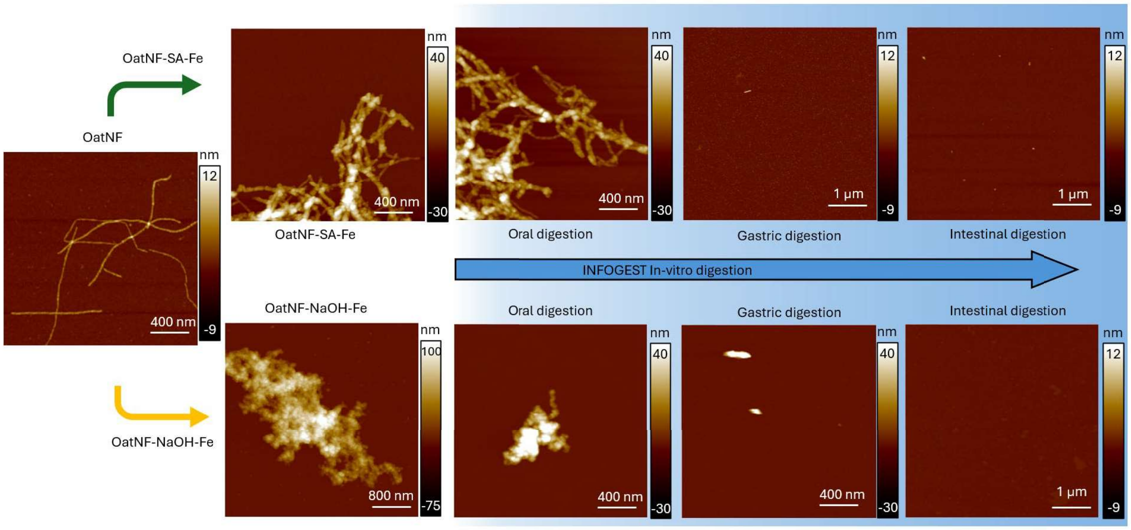

Supplementary Figure 5 | In-vitro gastrointestinal digestion of the OatNF-SA-Fe and OatNF-NaOH-Fe compound via AFM analysis. AFM images of OatNF, OatNF-SA-Fe and OatNF-NaOH-Fe during in-vitro gastrointestinal digestion. The time and phase dependent fates of OatNF-SA-Fe and OatNF-NaOH-Fe during digestion via the INFOGEST protocol are indicated by the arrow. Nanofibrils and iron particles were significantly digested during gastric digestion and the remainings were fully degraded in the subsequent intestinal digestion.

Supplementary Figure 6

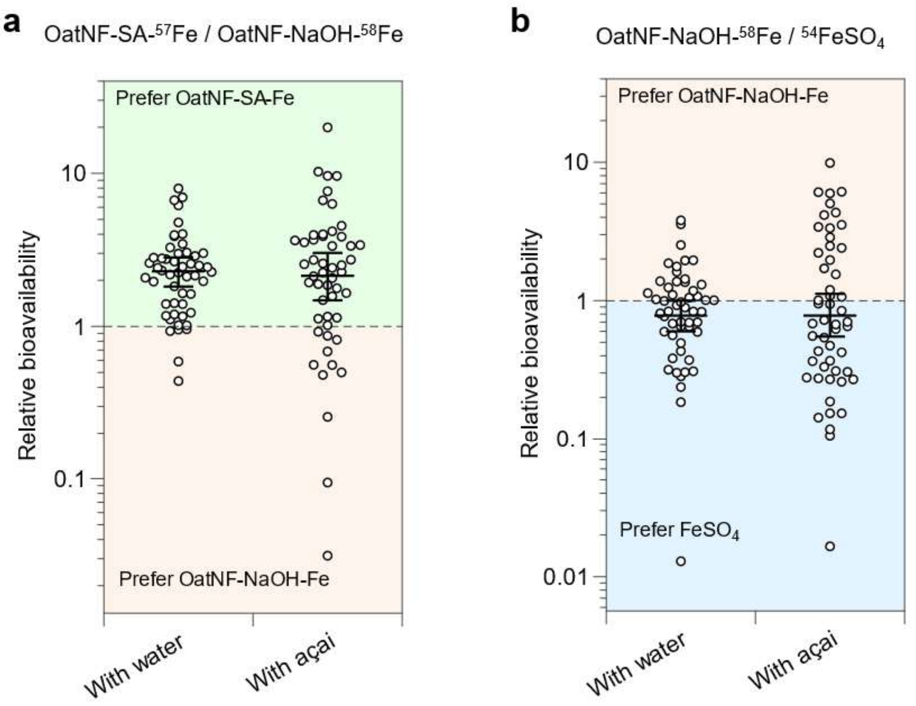

Supplementary Figure 6 | The relative bioavailability among OatNF-SA-^{57}Fe hybrids, OatNF-NaOH-^{58}Fe hybrids and ^{54}FeSO4 according to clinical study. The relative bioavailability between OatNF-SA-^{58}Fe hybrids and OatNF-NaOH-^{58}Fe (**a**); between OatNF-NaOH-^{57}Fe hybrids and ^{54}FeSO4 (**b**). The plots show means with whiskers showing SD (n=52 subjects).

Supplementary Table 1.

n	52
Age, y	32.0 ± 6.8
BMI, kg/m^2	21.5 ± 2.0
Haemoglobin, g/dL	12.80 ± 0.75
Serum ferritin, µg/L	31.4 (25.1 – 41.3)
C-reactive protein, mg/L	0.73 (0.47 – 1.46)

Data are mean ± SD or median (IQR).

Baseline characteristics of the women participating in the clinical absorption study.

Supplementary Methods

In-vitro INFOGEST digestion of the OatNF-SA-Fe and OatNF-NaOH-Fe hybrids

The INFOGEST protocol[5] was applied to simulate in-vitro gastrointestinal digestion of our compounds. The simulated salivary fluid (SSF), simulated gastric fluid (SGF) and simulated intestinal fluid (SIF) were prepared prior to the digestion. The digestion procedure was carried out at 37 °C under a gentle stirring condition to minimize the gelation. Immediately after digestion from each phase, the aliquots (10 µl) of sample were collected to prepare AFM samples by depositing on a freshly cleaved mica, followed by a gentle rinsing and drying process. AFM measurements were carried out by a Bruker multimode 8 scanning probe microscope (Bruker, USA) with a commercial silicon nitride cantilever (Bruker, USA) at a vibration frequency of 150 kHz. AFM images were flattened using Nanoscope 8.1 software (Bruker, USA) and no further image processing was applied.

Colorimetric assay

The ferrous and ferric iron content of dispersion was initially investigated by using potassium ferricyanide and potassium ferrocyanide assay. 4% potassium ferricyanide (CAS 13746-66-2, SigmaAldrich) and potassium ferrocyanide (CAS 14459-95-1, Sigma Aldrich) solution were prepared prior to the assay. An aliquot of diluted hybrid dispersion at different concentrations of $FeCl_3$ and SA in the plate were mixed with potassium ferricyanide and potassium ferrocyanide solution and measured the light absorbance (OD) in the microplate reader (Infinite M200PRO Tecan). The absorbances above detection limit are shown as n.a. in the plot.

The further determination of ferrous and ferric iron was achieved by colorimetric assay using ferrozine and ammonium thiocyanate, respectively. To study ferrous content, ferrozine (Sigma 160601) was added in diluted dispersion, reaching the final concentration of 100 µM. $FeSO_4$ and $FeCl_3$ solutions at different concentrations and Milli-Q water were used as control. After 10 min, the plate was transferred to the microplate reader (Infinite M200PRO Tecan) to measure the absorbance spectrum and light absorbance (OD) at 562 nm. To study the ferric iron, ammonium thiocyanate (Sigma 221988) was added into the diluted dispersion, reaching the final concentration of 300 mM. After 10 min, the plate was transferred to the microplate reader to measure the absorbance spectrum and light absorbance at 480 nm.

Supplementary References

1. Palika, A. *et al.* An antiviral trap made of protein nanofibrils and iron oxyhydroxide nanoparticles. *Nat. Nanotechnol.* **16**, 918–925 (2021).
2. Grosvenor, A. P., Kobe, B. A., Biesinger, M. C. & McIntyre, N. S. Investigation of multiplet splitting of Fe 2p XPS spectra and bonding in iron compounds. *Surface and Interface Analysis* **36**, 1564–1574 (2004).
3. Zhou, J. *et al.* Oat Plant Amyloids for Sustainable Functional Materials. *Advanced Science* **9**, 2104445 (2022).
4. Shen, Y. *et al.* Amyloid fibril systems reduce, stabilize and deliver bioavailable nanosized iron. *Nature Nanotech* **12**, 642–647 (2017).
5. Brodkorb, A. *et al.* INFOGEST static in vitro simulation of gastrointestinal food digestion. *Nat Protoc* **14**, 991–1014 (2019).
