# Supplementary material for: Oat protein nanofibril–iron hybrids offer a stable, high-absorption iron delivery platform for iron fortification
Source: Nat Food. 2025 Nov 10;6(12):1164–75. doi: 10.1038/s43016-025-01260-6 (PMC12717008; doi:10.1038/s43016-025-01260-6)
Supplement: Supplementary file 2 — Reporting Summary [file 43016_2025_1260_MOESM2_ESM.pdf]

nature portfolio

Corresponding author(s): Raffaele Mezzenga, Michael B. Zimmermann

Last updated by author(s): Sep 25, 2025

Reporting Summary

Nature Portfolio wishes to improve the reproducibility of the work that we publish. This form provides structure for consistency and transparency in reporting. For further information on Nature Portfolio policies, see our Editorial Policies and the Editorial Policy Checklist.

Statistics

For all statistical analyses, confirm that the following items are present in the figure legend, table legend, main text, or Methods section.

n/a	Confirmed	
☐	☒	The exact sample size (n) for each experimental group/condition, given as a discrete number and unit of measurement
☐	☒	A statement on whether measurements were taken from distinct samples or whether the same sample was measured repeatedly
☐	☒	The statistical test(s) used AND whether they are one- or two-sided<br>*Only common tests should be described solely by name; describe more complex techniques in the Methods section.*
☒	☐	A description of all covariates tested
☐	☒	A description of any assumptions or corrections, such as tests of normality and adjustment for multiple comparisons
☐	☒	A full description of the statistical parameters including central tendency (e.g. means) or other basic estimates (e.g. regression coefficient) AND variation (e.g. standard deviation) or associated estimates of uncertainty (e.g. confidence intervals)
☐	☒	For null hypothesis testing, the test statistic (e.g. F, t, r) with confidence intervals, effect sizes, degrees of freedom and P value noted<br>*Give P values as exact values whenever suitable.*
☒	☐	For Bayesian analysis, information on the choice of priors and Markov chain Monte Carlo settings
☒	☐	For hierarchical and complex designs, identification of the appropriate level for tests and full reporting of outcomes
☒	☐	Estimates of effect sizes (e.g. Cohen's d, Pearson's r), indicating how they were calculated

Our web collection on statistics for biologists contains articles on many of the points above.

Software and code

Policy information about availability of computer code

Data collection	TFS Talos F200X (Thermo Fisher Scientific, USA); JEOL JEM F200 (JEOL, Japan); SuperX EDS (Thermo Fisher Scientific, USA); TFS Titan Krios (Thermo Fisher Scientific, USA); PHI Quantera(Physical Electronics, USA); microplate reader (Infinite M200PRO Tecan), Neptune 3.3 (Thermo Fisher Scientific, USA), Nanoscope 8.1 (Bruker)
Data analysis	Casa XPS software; Origin Pro, Microsoft Excel 16.94; R version 4.4, IBM SPSS version 28, Nanoscope Analysis 8.1 (Bruker)

For manuscripts utilizing custom algorithms or software that are central to the research but not yet described in published literature, software must be made available to editors and reviewers. We strongly encourage code deposition in a community repository (e.g. GitHub). See the Nature Portfolio guidelines for submitting code & software for further information.

Data

Policy information about availability of data

All manuscripts must include a data availability statement. This statement should provide the following information, where applicable:

- Accession codes, unique identifiers, or web links for publicly available datasets
- A description of any restrictions on data availability
- For clinical datasets or third party data, please ensure that the statement adheres to our policy

The data in this paper are publicly available in the Supplementary Information and Source data provided with this paper. All other data are available upon the request to the corresponding authors.

Research involving human participants, their data, or biological material

Policy information about studies with human participants or human data. See also policy information about sex, gender (identity/presentation), and sexual orientation and race, ethnicity and racism.

Reporting on sex and gender	Our study included only female participants, as women are at higher risk for iron deficiency.
Reporting on race, ethnicity, or other socially relevant groupings	The study was conducted in Thailand, and the participants were recruited among students and staff of Mahidol University. The study population primarily reflects the demographic characteristics of university students and staff in Thailand. Data on race, ethnicity and socio-economic status were not collected.
Population characteristics	The population consisted of women aged 18-45 years, as women of reproductive age are most at risk for iron deficiency. Participants were selected based on their iron status (low iron stores with serum ferritin <50 ng/ml) but without anemia (Hb >12 g/dl)
Recruitment	The participants were recruited among students and staff of Mahidol University, Nakhon Pathom, Thailand, where the study was conducted.
Ethics oversight	The protocol of the study was approved by the Ethical Board of Mahidol University, Nakhon Pathom, Thailand

Note that full information on the approval of the study protocol must also be provided in the manuscript.

Field-specific reporting

Please select the one below that is the best fit for your research. If you are not sure, read the appropriate sections before making your selection.

☒ Life sciences ☐ Behavioural & social sciences ☐ Ecological, evolutionary & environmental sciences

For a reference copy of the document with all sections, see nature.com/documents/nr-reporting-summary-flat.pdf

Life sciences study design

All studies must disclose on these points even when the disclosure is negative.

Sample size	We based our power calculation on data from our previous iron absorption study that administered submicron-sized ferric phosphate in young women in Thailand19. In that study, the standard deviation observed between the differences of the logs of the fractional iron absorption (FIA) was 0.223. To resolve a difference of 30% in FIA with a power of 80% and a 5% error rate, we estimated the sample size to be 44 participants. To account for a 20% participant dropout, we recruited 52 participants.
Data exclusions	No data were excluded from the analysis.
Replication	Not applicable
Randomization	In each of the two study weeks, the order of the 3 conditions was randomized for each participant for each block using a Python script. The sequences were generated so that each isotope was used only once in each block.
Blinding	Blinding was not done; it is not necessary in single-meal iron absorption studies.

Reporting for specific materials, systems and methods

We require information from authors about some types of materials, experimental systems and methods used in many studies. Here, indicate whether each material, system or method listed is relevant to your study. If you are not sure if a list item applies to your research, read the appropriate section before selecting a response.

Materials & experimental systems

n/a	Involved in the study	
☒	☐	Antibodies
☒	☐	Eukaryotic cell lines
☒	☐	Palaeontology and archaeology
☒	☐	Animals and other organisms
☐	☒	Clinical data
☒	☐	Dual use research of concern
☒	☐	Plants

Methods

n/a	Involved in the study	
☒	☐	ChIP-seq
☒	☐	Flow cytometry
☒	☐	MRI-based neuroimaging

Clinical data

Policy information about clinical studies

All manuscripts should comply with the ICMJE guidelines for publication of clinical research and a completed CONSORT checklist must be included with all submissions.

Clinical trial registration	The study was registered at clinicaltrials.gov (ID No NCT05826899).
Study protocol	The study protocol is available from the Mahidol University Central Institutional Review Board (MU-CIRB) and/or the study investigators.
Data collection	The study was a single-center, prospective cross-over stable isotope trial, conducted at the Institute of Nutrition of Mahidol University, in Nakhon Pathom, Thailand.
Outcomes	The primary outcome was fractional iron absorption from the iron supplements. This was calculated based on the shift of the iron isotopic ratios in the collected whole blood samples, using the principles of isotopic dilution and assuming 80% incorporation of the absorbed iron into the erythrocytes.

Plants

Seed stocks	*Report on the source of all seed stocks or other plant material used. If applicable, state the seed stock centre and catalogue number. If plant specimens were collected from the field, describe the collection location, date and sampling procedures.*
Novel plant genotypes	*Describe the methods by which all novel plant genotypes were produced. This includes those generated by transgenic approaches, gene editing, chemical/radiation-based mutagenesis and hybridization. For transgenic lines, describe the transformation method, the number of independent lines analyzed and the generation upon which experiments were performed. For gene-edited lines, describe the editor used, the endogenous sequence targeted for editing, the targeting guide RNA sequence (if applicable) and how the editor was applied.*
Authentication	*Describe any authentication procedures for each seed stock used or novel genotype generated. Describe any experiments used to assess the effect of a mutation and, where applicable, how potential secondary effects (e.g. second site T-DNA insertions, mosiacism, off-target gene editing) were examined.*
